# Tolerance and heteroresistance to echinocandins in *Candida auris*: conceptual issues, clinical implications, and outstanding questions

**DOI:** 10.1128/msphere.00161-25

**Published:** 2025-04-16

**Authors:** Erika Shor, David S. Perlin, Dimitrios P. Kontoyiannis

**Affiliations:** 1 Center for Discovery and Innovation, Hackensack Meridian Health 721734, Nutley, New Jersey, USA; 2 Department of Medical Sciences, Hackensack Meridian School of Medicine 576909 , Nutley, New Jersey, USA; 3 Georgetown University Lombardi Comprehensive Cancer Center 66634, Washington, DC, USA; 4 Department of Infectious Diseases, Infection Control and Employee Health, The University of Texas MD Anderson Cancer Center 685136, Houston, Texas, USA; University of Georgia, Athens, Georgia, United States

**Keywords:** *Candida auris*, echinocandins, heteroresistance, tolerance

## Abstract

*Candida auris* is a significant public health threat due to its environmental persistence and multidrug resistance, with echinocandins being the preferred treatment. However, in addition to resistance, echinocandin tolerance and heteroresistance may contribute to treatment challenges. Echinocandin tolerance involves reduced drug-mediated killing, while heteroresistance is the ability of a small cell subset to grow at high drug concentrations. These phenomena may facilitate the emergence of full resistance and complicate clinical outcomes. The clinical significance of these mechanisms remains unclear, with limited data correlating them with treatment failures. Research is needed to understand their mechanisms and impact, develop streamlined and robust methods to detect them in clinical settings, and explore mitigation strategies. The pathogen’s range of drug adaptations demands innovative approaches like spatial transcriptomics to dissect these complex responses and improve patient outcomes.

## COMMENTARY

When *Candida auris* emerged as a new fungal pathogen over 15 years ago, it posed challenges similar to infections by other *Candida* species while exhibiting additional unique characteristics that rapidly made it a significant public health concern. Like other major *Candida* pathogens, *C. auris* forms robust biofilms on medical devices (e.g., catheters), shielding it from antifungals and immune attacks, contributing to persistent and often severe invasive infections, especially in highly vulnerable patients with comorbidities, frailties, and immunosuppression (1, 2). Additionally, it can manipulate host immune mechanisms, particularly innate immunity, to evade detection and hinder the body’s ability to clear infections (3). *C. auris* can also switch between different morphologies (yeast, filamentous forms, and pseudohyphae) to adapt to host environments (4, 5). Finally, it is capable of tremendous genetic plasticity, whereby it can rapidly evolve under stress, acquiring large-scale chromosomal changes (e.g., rearrangements and aneuploidies) and point mutations that enhance survival following exposure to antifungals and immune responses (6–8).

Moreover, in addition to all these already troublesome features that are shared by other *Candida* pathogens, *C. auris* possesses several distinct traits that further complicate its treatment and eradication. First, several clades of *C. auris* can exhibit resistance to multiple classes of antifungal drugs, including azoles, echinocandins, and polyenes, sometimes without a significant fitness cost in the host (9), leaving physicians with limited to no treatment options (10). Second, a distinctive phenotype exhibited by certain isolates of *C. auris* is their ability to form aggregates, and this has been linked to immune evasion, adhesion, and pathogenesis, even environmental persistence (11). We note that some debate remains regarding the role of cell aggregation in *C. auris* because heterogeneous results have been obtained depending on the experimental model and site of infection, with strain-to-strain variability and geographic origin/genetic clade as additional important confounders (12–17). Third, the high transmissibility of *C. auris,* coupled with the ineffectiveness of standard cleaning measures with many commonly used hospital disinfectants, including quaternary ammonium compounds, creates a “perfect storm” for its persistence in healthcare environments and its role in hard-to-control, widespread healthcare outbreaks (18). This is compounded by the fact that, unlike other *Candida* species, *C. auris* can survive for weeks to months on dry surfaces, contributing to spread (19, 20). Finally, *C. auris* exhibits unusually high tolerance to other stresses, such as high temperatures (up to 42°C) and high salt concentrations (20), enabling it to persist in environments with high salt concentrations, such as the skin.

*C. auris* high stress tolerance translates to its ability to withstand the action of multiple antifungal agents with different modes of action. Among the three major antifungal drug classes used for systemic infections, *C. auris* shows the lowest levels of resistance against echinocandins (ranging from 0% to 8%) (21), which are, therefore, the preferred treatment option. Echinocandins act by inhibiting the essential cell wall-building enzyme 1,3-β-glucan synthase and, unlike azoles, are fungicidal in *Candida* species. Echinocandin resistance (primary and acquired) has been described in multiple species, including *C. auris*, and is due to mutations in 1,3-β-glucan synthase *FKS* genes (22–24). However, in addition to frank resistance, defined as the ability to grow well above a certain drug concentration, two additional mechanisms have been recently identified *in vitro* and implicated as contributing to *C. auris* ability to survive in the presence of echinocandins—tolerance and heteroresistance. Both echinocandin tolerance and heteroresistance are characterized by increased fungal cell survival in the presence of what should be a fungicidal drug and are defined as follows.

### Echinocandin tolerance

Tolerance *in vitro* is generally thought of as a transient, non-genetically encoded phenomenon, and its definition depends on whether a drug is cidal or static. Antifungal drug tolerance has been predominantly studied in the context of azole antifungals, which are static in *Candida* species, and for which tolerance is usually defined as the ability to grow slowly above the minimal inhibitory concentration (MIC) (25–28). However, for cidal drugs such as echinocandins, a more appropriate definition is the one used for bactericidal antibiotics, in which tolerance is defined as reduced or delayed cell killing above the MIC (i.e., producing a killing curve with a shallower slope) (29). This slowed killing can be a characteristic either of a large part of the population or a small subset of cells, which are typically referred to as “persisters.” Although no *in vitro* echinocandin kill curve data for *C. auris* have been published, there is a strong suggestion that echinocandins might not be as cidal *in vivo*, i.e., in host tissues. For instance, in a murine model of systemic *C. auris* infection, treatment with various echinocandins significantly reduced but did not sterilize infected tissues (kidneys, hearts, and brains), resulting in residual drug-tolerant *C. auris* loads ranging from hundreds to millions of CFUs (30). Mechanistically, the limited *in vitro* studies on echinocandin tolerance in *C. albicans* have revealed a role in the transcriptional upregulation of cell wall stress responses, resulting in a compensatory increase in cell wall chitin levels (31). Furthermore, also in *C. albicans*, the acquisition of echinocandin or fluconazole tolerance has been associated with chromosomal alterations, specifically full or segmental aneuploidies (28, 31, 32). Another phenomenon, which may be thought of as a type of tolerance, is known as paradoxical growth or the “eagle effect,” whereby cells are inhibited at normal MIC concentrations but paradoxically are able to grow at supra-MIC concentrations. Although the paradoxical effect has been described for *C. auris* and echinocandins (22), its mechanisms, while likely associated with tolerance, are unknown.

### Echinocandin heteroresistance

Unlike tolerance, heteroresistance is defined similarly for static and cidal drugs as the ability of a small subset of the population to grow (e.g., form colonies) on a medium containing above-MIC drug concentrations. Because resistance is also defined based on growth at high drug concentrations, heteroresistance can be thought of as phenotypic resistance of a small subset of the population. In fungi, heteroresistance to azoles has been observed in several *Candida* and *Cryptococcus* species (33–36), and in *C. glabrata,* it was associated with an increased drug efflux capacity (36). On the other hand, heteroresistance to echinocandins (specifically, micafungin) has, thus far, been reported only in *C. parapsilosis* and *C. auris* (37). The mechanisms leading to echinocandin heteroresistance are not understood; because it is a phenomenon exhibited by a small subpopulation of presumably genetically identical cells, it may have an epigenetic basis. However, chromosomal number variations, i.e., aneuploidies, have been implicated in azole heteroresistance in *C. albicans* and *C. neoformans* (34, 35, 38).

The clinical significance of echinocandin tolerance and heteroresistance is unknown. Thus far, the role of fluconazole tolerance in therapeutic failures has been implied in *C. albicans* and *C. glabrata* (25, 27, 39), but no correlation between tolerance to echinocandins and their *in vivo* (36) efficacy has yet been reported. In *C. auris*, isolates exhibiting paradoxical growth in caspofungin *in vitro* have been examined in a mouse model of systemic candidiasis and were found to be as susceptible to caspofungin treatment as isolates incapable of paradoxical growth (22). Echinocandin heteroresistance was found to be associated with an increased risk of *C. parapsilosis* breakthrough bloodstream infections during micafungin prophylaxis in patients with allogeneic stem cell transplantation (37). However, it is still unclear if heteroresistance by itself would explain these breakthrough infections; *C. parapsilosis* notably has an innately elevated echinocandin MIC relative to other *Candida* species due to a naturally occurring *FKS1* hot-spot polymorphism, which makes the therapeutic window much narrower and may be an additional factor increasing the risk of breakthrough infections.

In Box 1, we list some outstanding basic, translational, and clinical research questions related to echinocandin tolerance and heteroresistance that would be valuable to address in *Candida* in general and *C. auris* in particular.

Box 1. **Outstanding questions regarding the translational and clinical implications of echinocandin tolerance and heteroresistance in *C. auris***

- Since more cells are alive and available to acquire genetic mutations both in echinocandin tolerance and heteroresistance, are these phenomena stepping stones for the emergence of full-blown resistance? If so, what are the molecular mechanisms?

- Are *Candida* strains that are echinocandin-heteroresistant also more echinocandin-tolerant and *vice versa*? If so, what are the molecular underpinnings?
- Are there any fitness trade-offs associated with echinocandin tolerance and/or heteroresistance?
- How much variability in frequency and magnitude of echinocandin tolerance and/or heteroresistance exists within strains and various *Candida* species that exhibit conventional susceptibility or non-susceptibility to echinocandins?
- Are there echinocandin-specific (caspofungin, micafungin, and anidulafungin) differences in frequency and magnitude of echinocandin tolerance and/or heteroresistance?
- Does exposure of *Candida* to a new generation of echinocandins with long half-life (i.e., rezafungin) and high *C*_max_/MIC alter the frequency and magnitude of echinocandin tolerance and/or heteroresistance?
- Is there an association between echinocandin tolerance and/or heteroresistance and *Candida* morphogenesis (as it has been described with *C. auris* tolerance to azoles [40]) and virulence?
- Could one expect tolerance and/or heteroresistance with new first-in-class agents that have novel mechanisms of action (e.g., fosmanogepix)?
- As monotherapy induces stress adaptation, would combinations of two cidal drugs (e.g., amphotericin B and echinocandins) or other antifungal adjuvants (e.g., chloroquine or nikkomycin) abrogate echinocandin tolerance and/or heteroresistance (41–43)?
- Can artificial intelligence be used to “predict” tolerance and resistance based, for example, on clinical parameters and whole-genome sequencing of *Candida* isolates (37)?
- Can methods be developed to detect, in a time-efficient and practicable way, echinocandin tolerance and/or heteroresistance in the clinical laboratory (33)?
- Can highly multiplexed spatial single-cell transcriptomics, as recently described in bacteria (44), dissect the complex mechanisms of diverse and heterogeneous responses of *C. auris* to echinocandins on a population level?
- Is echinocandin tolerance and/or heteroresistance in *Candida* more prevalent in complex polymicrobial environments (e.g., in biofilms or gut mucosa)?
- Can optimization of echinocandin pharmacokinetics in settings associated with lower drug exposures (e.g., gastrointestinal mucosal surfaces), high volume of distribution in critically ill patients in ICU (45), and biofilms mitigate echinocandin tolerance and/or heteroresistance?

In conclusion, the responses of *Candida* to a cidal drug such as an echinocandin might be more complex and heterogeneous than previously thought, and this is certainly the case for the multidrug-resistant emerging pathogen *C. auris*. Those dynamic and diverse responses are probably amplified in the complex *in vivo* microenvironment of tissue infections (e.g., various and fluctuating iron and nutrient availability, O_2_/CO_2_ concentrations [46], immune cells, albumin, polymicrobial communities, and biofilms) and cannot be easily captured by current conventional *in vitro* tests. As many population-level susceptibility phenotypes in *Candida* are driven by heterogeneous responses adapted by single cells, more efforts are needed to develop sensitive, high-throughput multiplex spatial transcriptomic methods (44) to dissect those phenotypes and uncover the metabolic strategies driving these phenotypes. Clinicians should be reminded that *Candida*, especially *C. auris*, is a highly sophisticated and nimble pathogen with tremendous adaptability and capacity to resist killing.

Finally, the emerging complexities of phenotypes *in vitro* as relating to the *in vivo* capability of cidal drugs such as the echinocandins underscores the complexities of *in vitro–in vivo* correlations and outcomes in candidiasis (47). Typically, failure of an antifungal is multifactorial, MICs do not reflect the whole story (47), and heteroresistance and/or tolerance, which are nearly impossible to detect in most clinical microbiology labs, might underlie some of these failures despite apparently “low” MICs. As is the case in infection in general, pharmacokinetics/pharmacodynamics play key roles (48), especially for pathogenic species such as *C. auris* that have innately high MICs and propensity for tolerance/heteroresistance. In the big picture, within the setting of persistent infection and in view of the tremendous genomic versatility of *Candida*, a range of echinocandin non-susceptible phenotypes might be operative, which can be associated with enhanced virulence traits (immune evasion properties, biofilm formation, and pathogenicity), as it has been shown recently in patients with refractory *C. albicans* fungemia (49).
